# Leptin and Asthma: What Are the Interactive Correlations?

**DOI:** 10.3390/biom12121780

**Published:** 2022-11-29

**Authors:** Yang Wang, Chengping Hu

**Affiliations:** 1 Department of Respiratory Medicine (Department of Respiratory and Critical Care Medicine), Xiangya Hospital, Central South University, Changsha 410008, China; 2 National Clinical Research Center for Geriatric Disorders, Xiangya Hospital, Central South University, Changsha 410008, China

**Keywords:** leptin, asthma, obesity, inflammation

## Abstract

Leptin is an adipokine directly correlated with the proinflammatory obese-associated phenotype. Leptin has been demonstrated to inhibit adipogenesis, promote fat demarcation, promote a chronic inflammatory state, increase insulin sensitivity, and promote angiogenesis. Leptin, a regulator of the immune response, is implicated in the pathology of asthma. Studies involved in the key cell reaction and animal models of asthma have provided vital insights into the proinflammatory role of leptin in asthma. Many studies described the immune cell and related cellular pathways activated by leptin, which are beneficial in asthma development and increasing exacerbations. Subsequent studies relating to animal models support the role of leptin in increasing inflammatory cell infiltration, airway hyperresponsiveness, and inflammatory responses. However, the conclusive effects of leptin in asthma are not well elaborated. In the present study, we explored the general functions and the clinical cohort study supporting the association between leptin and asthma. The main objective of our review is to address the knowns and unknowns of leptin on asthma. In this perspective, the arguments about the different faces of leptin in asthma are provided to picture the potential directions, thus yielding a better understanding of asthma development.

## 1. Introduction

Asthma is a chronic heterogeneous inflammatory airway disease with various cellular component recruitments associated with reversible airway obstruction and respiratory symptoms, such as wheezing, cough, and shortness of breath [1,2]. Multiple pathogenic mechanisms of asthma were poorly elucidated [3]. Obesity, considered to be with a standard body mass index (BMI) of ≥30 kg/m^2^, has shown an increasing prevalence in recent years [4]. The low-degree state of systemic inflammation in obesity, involving the activation of M1 macrophages, and CD8^+^ T lymphocytes, could produce multiple inflammatory agents, including IL-1β, IL-6, IFN-γ, and TNF-α [5]. Previous studies have implicated that obesity is related to asthma severity, and most obese patients with asthma respond poorly to conventional treatment (corticosteroids) [6,7]. Obesity has been correlated with the severity of asthma [8,9], the multiple mechanisms of which are relevant to genetic, hormonal, environmental, mechanical, and immunological factors [10]. There are two hypotheses about the relationship between obesity and asthma: one is diaphragm excursion due to fat deposit and limited thoracic compliance [10], and one is the immunological and inflammatory adipokines derived from adipose tissue, such as leptin and adiponectin [11]. It is well established that chronic obesity shifts M2-polarized macrophages to M1-polarized macrophages in adipose tissue [12]. It was proposed that airway hyperresponsiveness (AHR) in an obese asthma mouse model was mediated by adipokines and inflammatory cytokines (TNF-α, TGF-β, IL-1β, and IFN-γ), which had a poor response to dexamethasone. While AHR in a lean asthma mouse model was mediated by eosinophils, Th2 cells, and Th2-related cytokines (IL-5, IL-4, and IL-13), which could be reversed by dexamethasone [13]. Among the numerous adipokines secreted by adipocytes, the level of leptin in the serum of obese people is significantly higher than that in non-obese population [14,15]. Leptin is a 16KD product of the obese gene (ob), which acts as a factor contributing to inhibiting adipogenesis, promoting fat demarcation, promoting a chronic inflammatory state, increasing insulin sensitivity, and promoting angiogenesis [16,17,18]. Obesity was reported to aggravate the severity of asthma accompanied by several comorbidities, such as obstructive sleep apnea (OSA) and hypertension [19]. OSA, a kind of obesity-related sleep and breathing disorder, is known to be associated with increased leptin secretion [20,21]. OSA is usually accompanied by asthma, and they have many common risk factors, such as intermittent hypoxia, inflammation, leptin, and obesity [22]. Apart from obesity, OSA can also influence airway inflammation in asthma due to complex oxidative stress induced by repetitive hypoxia [23,24]. The use of continuous positive airway pressure (CPAP) is a preferred treatment for OSA [25]. CPAP treatment could improve hypoxemia and decrease inflammatory markers of OSA, such as CRP and IL-6 [26]. However, the effect of CPAP on leptin levels is controversial. Some studies indicated that the effect of CPAP therapy on leptin levels of OSA patients is limited [27,28,29]. While another study showed that hyperleptinemia of OSA patients could be normalized by the therapy with nasal CPAP [30]. Thus, more studies are required to explore the interaction between CPAP and leptin levels in OSA patients. Remarkably, weight loss is the effective therapy way for OSA and asthma [31].

Compared with the studies that referred to leptin and diabetes or obesity, very few studies were associated with leptin and asthma, and the mechanistic basis for the role of leptin in asthma has not been established completely. The objective of our study is to review the present data supporting the pathological role of leptin in asthma, including studies from clinical cohort studies and animal models.

## 2. Physiological Role

### 2.1. Main Roles of Leptin

Leptin, a protein composed of 167 amino acids, is primarily produced by fat cells and macrophages in adipocytes [32]. Leptin is distributed in the lung, including alveoli Type II pneumocytes, macrophages, and so on [33,34]. Leptin has several faces as a hormone with the function of regulating food intake and energy expenditure and producing pro-inflammatory cytokines [35]. Leptin has structural homology with such cytokines as interleukin-6 and interleukin-11, implying an effect of immunomodulating [36]. The cis-elements, with sequences distributing between −22 kb and +150 kb, were reported to be required for leptin gene expression [37]. NF-Y is a CCAAT-box binding transcription factor consisting of three subunits (NF-YA, NF-YB, and NF-YC). These three subunits are identified to have DNA binding activity, and the CCAAT sequence is recognized by NF-Y through the conserved C-terminus [38]. It was suggested that the corresponding sequences of the leptin gene were recognized by NF-Y enhancer at −16.5 kb, and loss of NF-Y contributed to hypoleptinemia and lipodystrophy [39]. Previous papers reported that leptin could promote adipocytes to secret pro-inflammatory cytokines, such as TNF-α, IL-6, and IL-12 [17,40,41]. Several studies indicated that inflammatory cytokines (tumor necrosis factor (TNF), IL-1, and LPS) and hypoxia could induce leptin production from adipocytes [42,43,44,45] and promote allergic airway responses in mice [22,46,47].

Obesity is related to the high level of leptin, as well as the anorectic resistance of leptin [48,49]. The level of serum leptin in obese people is 4–6 times higher than that of non-obese people [50], especially in women [51]. Besides, obese patients develop leptin resistance. Thus the increased leptin levels no longer regulate satiety, and the hypothalamus showed insensitivity to leptin [52,53], the mechanism of which may be the direct action of leptin [54,55]. It was shown that compared with non-obese asthmatic mice, higher leptin levels were observed in obese asthmatic mice [56]. Leptin was reported to be related to body weight-gain-related asthma by regulating lung injury [57,58]. OVA-treated mice have been shown to elevate serum leptin levels, whereas exogenous leptin administration increased OVA-induced AHR and serum IgE levels [59]. Besides, leptin exerts distinct effects on viral infection. The ob/ob mice (leptin deficiency mice) infected with the encephalomyocarditis virus resulted in a more severe myocardial injury via elevating TNF-α expressions in comparison with wild-type mice [60]. Similarly, db/db mice (leptin receptor deficiency mice) were more susceptible to the infection of Coxsackie virus B4 than wild-type mice [61]. In RSV-infected human bronchial epithelial cells, the oversecreted leptin facilitated Th17 cell differentiation but inhibited Th2 cell differentiation via modulating ERK1/2 phosphorylation [62]. Viral infections have been implicated in asthma development. Obese mice with low survival rates had low cytotoxicity of the natural killer cells after the infection of the influenza virus [63]. In this regard, there may be a vital role of leptin in asthma with virus infection.

### 2.2. Mechanism of Action

Leptin exerts its function by binding to the leptin receptor (Ob-R), a product of the diabetes (db) gene [64]. Ob-R, which is widely expressed in immune cells, is a member of the superfamily of class I cytokine receptors (gp130) [65]. There are at least six isoforms produced by alternative splicing of ob-R, containing the same N-terminal binding domain but different cytoplasmic domain lengths of ob-R isoforms: ob-Ra, ob-Rb, ob-Rc, ob-Rd, ob-Rf, and ob-Re [66,67]. Ob-Rb was reported to be the isoform that transduces the downstream signaling [68]. Besides, four splice variants of obR have been identified in humans: a long isoform responsible for most functions of leptin and three short isoforms [69,70]. The short isoforms cannot transduce hormone signals. Only the long isoform (ob-Rb) can signal correctly [71]. Leptin and ob-Rs are expressed in epithelial cells, type II alveolar cells, and macrophages of the lung [33,34,72,73,74]. Leptin, bound to the leptin receptor, could produce cytokines and enhance proliferative responses by activating the pathways of MAPK, JAK2-STAT3, and PI3K-AKT [17,75,76,77,78,79]. In another study, it was mentioned that LEP polymorphism of the leptin 5′-UTR (rs13228377) was related to high leptin levels in asthma, while polymorphisms of leptin receptors (K109R and Q223R) did not show a significant correlation with serum level of leptin receptors [72]. In a logistic regression analysis, rs13228377 polymorphism of leptin and leptin level showed good predictive accuracy, indicating increased asthma risk [72]. Moreover, a previous study showed that the Gln223Gln genotype of the leptin receptor was implicated in lower binding capacity to leptin, which may be the mechanism of leptin resistance [80,81].

Leptin was involved in the activation, differentiation, and proliferation of immune cells [75,76]. It has been reported that leptin could promote Th1 responses in vivo but displayed discrepant effects on Th2 responses [78,82,83]. Several studies have demonstrated that leptin/IL6 signaling plays a critical role in inflammatory responses through activating STAT3, ultimately resulting in the pathogenesis of asthma [58,84]. It was reported that exogenous administration of leptin increased the airway hyperresponsive of the asthma mouse model [59]. OVA challenge elevated serum leptin production in mice, particularly in leptin-infused mice, while leptin has no significant effect on airway responsiveness and IgE production without allergic airway challenge [59]. Leptin increased the lung resistance, tissue damping, and fibrosis markers in HDM-treated mice, but not leptin or HDM alone, with more effects observed in female mice than in male mice [85]. In line with the previous studies, leptin acts on macrophages or lymphocytes activated by other moieties; the effect is absent with leptin alone [17,59,78,79].

## 3. Leptin and Asthma

### 3.1. Epidemiological Studies

Among these studies, high leptin was identified frequently in patients with asthma [72,86,87,88,89]. Some studies have reported inverse correlations between leptin and lung function [88,90,91,92] as well as weight loss [88,91,93]. There were positive associations between leptin and symptomatic atopy [89] and BMI [86,94]. While, some studies reported that there was no relationship between leptin and lung function [93,95] and BMI [96].

One longitudinal study followed pulmonary inflammatory markers, lung function, and asthma activity of obese asthma patients undergoing bariatric surgery. In a population of 19 patients with asthma, the study manifested a reduction in systemic levels of leptin and an improvement in asthma activity scores after bariatric surgery through 1-year follow-up [93]. Another longitudinal study followed leptin levels, lung function, exercise-induced bronchospasm, and asthma-related symptoms of post pubertal obese adolescents undergoing weight loss of interdisciplinary intervention. In a population of 84 obese adolescents, the study showed a reduction in leptin levels and asthma symptoms after weight loss through 1-year therapy [91]. However, the above-mentioned studies did not include a control group, limiting further extrapolations.

Systemic leptin levels were found to be increased after the systemic exogenous glucocorticoid administration [97,98]. At the same time, a longitudinal study indicated that higher serum leptin was observed in asthmatic children before budesonide treatment than after budesonide treatment or control group. The difference in leptin levels between patients with asthma and without asthma was not significant after budesonide treatment in 4 weeks. Moreover, serum leptin levels correlated with body mass indices after budesonide treatment rather than before budesonide treatment [99]. The inhaled corticosteroids exhibited few systemic bioavailabilities, and leptin decreased after budesonide treatment may attribute to reduced airway inflammation and T-cell responses due to inhaled steroids rather than body weight and lipid metabolism [99].

The cross-sectional studies manifested some findings, from high levels of leptin in overweight patients with asthma compared to normal weight patients with asthma or in asthma patients of obese compared to non-obese asthma patients [86] to high levels of leptin related to BMI and severe lung function [86,88,90,94]. While some studies revealed no differences in leptin between overweight patients with asthma and without asthma [94] or no differences between leptin and lung function or BMI [93,95,96]. There were a series of studies revealing the positive correlations between leptin and asthma severity [72,90,96,100], suggesting leptin could be used as a pro-inflammatory biomarker in severe asthma. An interesting study of the leptin level after calorie restriction and weight loss was carried out by James et al. [101]. Nine of the subjects lost an average of 8% weight during an alternate day restricted calorie (ADCR) dietary regimen, including eating ad libitum (AL) and alternate day restricted calorie (CR) dietary regimen. It was revealed that lower leptin levels were observed on CR days compared with AL days, and leptin levels showed a decreasing level on AL days at 8 weeks. Moreover, the asthma symptoms and PEF improved through the study [101]. The correlations between leptin level and BMI or uncontrolled asthma score were more evident among female patients with asthma [86]. The controversial results between these findings may be associated with the variation of age, race, and gender in leptin. Ten cross-sectional studies and five longitudinal studies were searched by reviewing the PubMed database (Table 1).

### 3.2. Mechanistic Studies

From the extensive literature about the cellular role of leptin, we reported studies to address the pathophysiology of asthma.

#### 3.2.1. Airway Epithelial Cell Dysfunction and Mucus Secretion

It is important to keep the integrity of the bronchial epithelial natural barrier against allergens or pathogens [102]. The dysfunction of airway epithelial cells could promote the evolution of asthma exacerbation. It has been proposed that Ob-R was present in human bronchial and alveolar epithelial cells [103,104]. Leptin, binding to obR, directly activates the migration of human airway epithelial cells, inhibits apoptosis, promotes proliferation, and enhances the production of CCL11, VEGF, G-CSF, and IL-6 in a dose-dependent manner. However, the administration of Ob-R siRNA abolished the expressions of CCL11, and ICAM-1 induced by leptin [105]. Leptin has been shown to augment VEGF production, which is the key substance in airway remodeling [106,107]. Leptin inhibited the proliferation, migration, and eotaxin production in IL-13-induced human airway smooth muscle, while leptin did not promote airway smooth muscle cells to produce proinflammatory cytokines [104,108]. Besides, leptin regulates the secretion of MUC5AC in IL-13-induced human bronchial epithelial cells, which is a vital component of airway mucus [109].

#### 3.2.2. Immune Cell Responses

Immune cell activation is of great significance in asthma development. The obese OVA mouse group showed a higher neutrophil number at 48 h and higher macrophage numbers at any time, a higher arginase-positive rate of macrophages at 24 h, a higher rate of iNOS-positivity at 48 h, lower eosinophil numbers in the pulmonary tissue, lower levels of IgE, higher numbers of mast cells, and higher goblet cell hyperplasia in comparison with the lean-with-OVA group after the last OVA challenge [110]. Leptin has also been found to play a regulatory role in the immune system [111]. High leptin level was reported to inhibit neutrophil death by activating MEK1/2 and NF-κB pathways, produce neutrophil chemotaxis by activating ERK1/2 and p38-MAPK pathways; stimulate natural killer cells and macrophages to release inflammatory factors, and promote epithelial cells and smooth muscle cells in the airway to proliferate in asthma [112,113]. As mentioned above, exploring the correlations between leptin and immune cells is a prerequisite for a better understanding of the downstream mechanisms of asthma.

##### Lymphocyte Cells

Previous studies have indicated that leptin promotes T-cell proliferation and activation [14,114]. High levels of serum leptin increased immune cell activation in the obese state, thereby activating pro-inflammatory Th1 cells [115,116]. Leptin promotes Th1 cell activation while suppressing the Th2-related cytokine levels (IL-4, IL-5, and IL-10) [77,78,115,117]. Leptin modulates T cells to a Th1 immune response by increasing IFN-γ production [118,119]. When leptin is deficient, the Th1 phenotype is shifted to the Th2 phenotype, followed by the reduction in the total number of CD4^+^T cells was reduced [120,121]. Leptin, via binding to leptin receptors, was found to convert CD4^+^ T lymphocytes into Th1 cells [77,78,118]. While another study showed that leptin promoted Th2 cell proliferation but not Th1 cells under the condition of type 2 responses [122]. A recent report indicated that STAT3 activated by leptin was required for the IL-6-mediated anti-apoptotic T cell function [123]. Leptin also activates pro-inflammatory Th17 cells [124]. Leptin elevated Th17 cytokine levels while reducing the function of Treg cells in the culture of CD4^+^ T cells from lean allergic-asthma patients [121,125]. Leptin shifts T-helper (Th) cells to Th1 cells by producing IFN-γ [126]. Moreover, Th1/Th17 lymphocytes could secrete leptin, which in turn potentiates its effects on Th1/Th17 differentiation [78]. Besides, leptin promotes naive T cell and memory T cell proliferation and inhibits the proliferation of CD4^+^ CD25^+^ regulatory T cells [64,127]. Leptin promotes naïve T cells or effector T cells proliferation while suppressing memory T cells and Tregs [127,128].

##### Macrophages

It was suggested that macrophages in the sputum of obese patients with asthma were increased, as compared with non-obese patients with asthma. The markers of M2 macrophages were reduced in the sputum of obese patients with asthma, the mechanisms of which may be increasing oxidative stress and impaired response to corticosteroids [129]. In comparison, it was noted that M1 macrophages were elevated in an obese asthma mouse model compared to a nonobese mouse model [130]. While few studies have indicated the associations between leptin and M1 macrophages in an obese asthma mouse model. It has been shown that leptin is a chemoattractant for monocytes/macrophages by activating the obR long-form receptor and PI3K signaling [131]. Leptin elevates the phagocytic function of macrophages/monocytes and leukotriene synthesis production in pulmonary K. pneumoniae infection [132]. Leptin could stimulate monocytes and macrophages to produce inflammatory cytokines (TNF-α, IL-6) and reactive oxygen species via activating the Ob-Rb [76]. In addition, leptin was suggested to elevate the production of TNF-α and IL-6 in LPS or ozone-stimulated macrophages [133,134]. Leptin could promote human peripheral blood mononuclear cell (PBMC) proliferation, increasing the response of monocyte to LPS and stimulating cytokine secretion (IL-6 and TNF-α) [135,136]. Leptin could enhance leukotriene synthesis in alveolar macrophages, which may lead to bronchoconstriction [137]. An in vitro study revealed that leptin could induce the production of IL-6 and TNF-α in PBMC in a dose-dependent manner [138]. However, it was indicated that leptin acted on macrophages and lymphocytes only when leptin and other substances worked together, not alone [17,78,79].

##### Neutrophils and Eosinophils

It has been suggested that neutrophils are related to the severity of asthma [139], especially in obese asthma patients with severe symptoms [140,141]. Neutrophils are critical immune cells leading to asthma development and glucocorticoid resistance by producing chemokines, cytokines, and MPO granules [142]. Leptin can exacerbate airway inflammation by recruiting eosinophils and neutrophils [112,143]. Leptin contributes to neutrophil accumulation at the sites of inflammation [144] and modulates neutrophil chemotaxis [145,146]. Besides, leptin has been noted to induce eosinophil and neutrophil chemotaxis through activating ERK1/2 and p38-MAPK signaling, to inhibit neutrophil death through activating of MEK1/2 and NF-κB signaling pathway, resulting in advanced airway inflammation and remodeling [112,147]. Leptin cannot directly activate neutrophils but could be a chemoattractant for neutrophils, and recent studies have shown that physiological concentrations of leptin are not sufficient to induce the effects of leptin in neutrophils [130,131,137,148]. Besides, recent reports indicated the LEPR short receptor (ob-Ra) expressed in polymorphonuclear neutrophils (PMNs) activated the MAPK pathway and ROS production [69,145]. Besides, leptin delays neutrophil apoptosis at high concentrations in vivo and in vitro [131,149]. The bacterial phagocytic function of neutrophils is impaired when leptin is absent, and the administration of leptin could reverse the response, which is mediated by the complement receptors [150].

Leptin appeared to be a survival cytokine for human eosinophils [151]. Leptin could induce eosinophil chemotaxis, which is associated with increased calcium mobilization [112]. Conus et al. reported that leptin delayed eosinophil spontaneous apoptosis via acting on ob-Rb [151], and leptin increases the antiapoptosis survivin and baculoviral IAP repeat containing 5 (BIRC5) might be the underlying mechanism [152,153]. Johnston et al. reported a lower level of eosinophils in the bronchoalveolar lavage fluid was observed in an ob/ob mice asthmatic model compared with the wild-type asthmatic model [154]. Recently, Wong et al. indicated that leptin promoted eosinophil migration by activating the MAPK pathway and induced eosinophils to produce inflammatory cytokines (IL-1β, IL-6, IL-8, GRO-α, and MCP-1) [155]. However, the role of leptin in neutrophil and eosinophil activation remains to be explained in obese asthma mice.

##### Other Immune Cells

Dendritic cells (DCs) have the function of antigen-presenting in the immune systems. Leptin could upregulate the markers of activated human DCs, such as TNF-α, IL-1β, IL-6, and MIP-1α, and prepares them for Th1 differentiation [156,157], the mechanism of which may be inhibiting apoptosis [158]. It was reported that leptin-activated DCs by promoting glycolytic metabolism and the STAT3-HK2 pathway [159]. DCs showed higher efficiency in inducing Treg or Th17 cells in the absence of leptin than in the presence of leptin [160].

Type 2 innate lymphocytes (ILC2s) have been demonstrated to be the responders in the early stage of the experimental asthma mouse model induced by various agents [161,162]. The numbers of ILC2 were decreased under the condition of leptin deficiency, ultimately resulting in the alleviation of asthma [122].

Mast cell influx is involved in allergic airway inflammation of obesity, resulting in delayed immune response and promoting asthma severity [110]. Leptin was reported to promote pro-inflammatory mast cells to degranulation and secrete histamine by inducing the release of intracellular Ca^2+^ and chemokine CCL3 [163].

In general, several immune cells are involved in leptin-mediated immune responses. The conflicting modulation of Th2 cells implies the effect of leptin is probably influenced by some extrinsic and intrinsic factors. Related mechanisms should be further explored in obesity-related asthma with some experimental studies. Besides, more experimental data are required to validate the effects of leptin on dendritic cells and mast cells in obesity-related asthma. The correlations between leptin and immune cells are summarized in Table 2.

## 4. Obesity-Associated Asthma

Several studies indicated that obese asthma patients occurred more frequently in mild to severe, female, non-allergic, late-onset asthma patients, with symptoms difficult to be controlled, impaired lung function, and frequent exacerbations than non-obese patients with asthma [164,165,166]. Obesity increases systemic inflammatory cytokines and immune cell recruitment [167]. Moderate obesity is accompanied by weight and fat gain, metabolic disturbances, and low-grade systemic inflammation [168]. The underlying mechanisms of the association between asthma and obesity have not been fully elucidated. There were several hypothesized possibilities for the pathogenesis of obesity and asthma [169], including genetic and environmental factors [170], lung volume and airway diameter reduction in obese individuals [171], obesity comorbidities such as sleep-disordered breathing [172], and last but not least, chronic obesity systemic low-grade inflammation [173]. Meanwhile, some reports showed that the status of obesity systemic low-grade inflammation was reported to elevate the levels of cytokines, chemokines, and leptin in the serum [174,175]. Obese mice with ovalbumin sensitized and challenged exhibited lower eosinophil numbers in BAL fluid at 24 h and 48 h, while higher eosinophil numbers at 72 h, higher eosinophil infiltration in the bronchiolar segments, and higher levels of interleukin (IL)-5, TNF-α, and IL-10 in BAL fluids than modeled lean mice [176]. Total numbers of macrophage and eosinophil in BALF, AHR, and eosinophilic inflammation in the histopathological analysis were increased in the OVA-obese group compared with the OVA-lean group. However, no significant differences were observed in the unmodeled obese and lean groups [59,177].

Recently, several studies showed that leptin was related to advanced asthma symptoms and airway hyperresponsiveness. However, few studies have demonstrated the effects of leptin on obese asthmatic patients regarding airway inflammation [91,92]. Previous studies about animal experiments have tried to decipher the relationship between leptin and obese asthma. The high-fat-fed mice showed increased body weight, lipid profile alterations, and high serum leptin compared with the lean mice [176]. The expressions of leptin and leptin receptors in obese mice were enhanced compared to mice in the control group but had no difference between obese mice and obese asthma mice [178]. Leptin elevated the airway resistance in OVA-sensitized/challenged mice but had no apparent influence on unmodeled mice, suggesting that leptin synergized with other substances to act rather than alone [59]. It has been revealed that obesity-associated hyperleptinemia enhanced the levels of the unfolded protein response factor XBP1s to elevate Th2 responses, leading to asthma exacerbation [179]. Obese asthmatic mice sensitized and challenged by OVA showed higher serum leptin levels, a higher number of neutrophils and lower numbers of macrophages in BALF, and more severe inflammation of the airway in comparison with non-obese asthmatic mice. At the same time, simvastatin could reverse the changes in obese mice. Moreover, the neutrophil percentage in BALF had a positive correlation with serum leptin levels [56].

Notably, a recent study has reported that OVA challenge in ob/ob mice (leptin-deficient obese mice) elevated the infiltrated eosinophil in the lung and enhanced the levels of TNF-α and IL-10 in BAL fluids while emigrating lower eosinophil in BAL fluids, and reduced IL-6 levels in comparison with OVA challenge WT mice [180]. Obese-OVA mice showed more severe airway inflammation, higher eosinophils in BALF, and higher leptin level than non-obese OVA mice [177]. Roflumilast (a PDE-4 inhibitor) was reported to ameliorate the eosinophil proliferation of BALF cells and serum levels of leptin in obese OVA mouse models [13]. At the same time, the administration of IL-17 inhibitor reduced airway inflammation and the leptin/adiponectin ratio in the obese-OVA mice [177]. Eosinophil airway inflammation induced by IL-33 was decreased in ob/ob-modeled mice compared with WT-modeled mice. In comparison, the administration of exogenous leptin reversed the changes of IL-33-induced in ob/ob modeled mice [181].

Studies relating to animal models support the role of leptin in obesity asthma. However, most animal studies are based on eosinophil asthma, and few studies explored the role of leptin in neutrophil asthma mouse models, although obese asthma was seen more frequently in non-atopic asthma. Furthermore, future studies are suggested to demonstrate the role of leptin with more regulatory mechanisms in obese-associated airway inflammation in the asthma mouse model, and whether there is a regulatory feedback mechanism remains unclear.

Weight loss was reported to reduce circulating leptin concentration [182,183] and improve the symptoms of asthma [184,185]. Reduced levels of leptin after moderate or massive weight loss were a predictor for lung function improvements in obese adolescents [91]. Bariatric surgery (BS) led to a significant weight loss at 12 months. FEV1, total lung capacity, functional residual capacity, asthma control, and systemic inflammation markers (CRP and leptin) were improved in the asthma group with BS [186]. Maniscalco et al. found that asthma control was improved after weight loss in women patients undergoing bariatric surgery [187]. Johnson et al. reported oxidative stress markers were reduced after the caloric restriction in asthma patients [101]. While another study reported that dietary-induced weight loss failed to improve airway hyperreactivity in patients [188]. Altogether, these results indicate that weight control needs to be considered in the treatment of asthma with obesity.

## 5. Conclusions

Leptin is implicated in the pathophysiological and cellular mechanisms of the development of asthma. Studies involved in the cellular immune responses and asthma animal models have provided vital insights into the deleterious role of leptin in asthma. Epidemiological studies mainly demonstrated the correlation of leptin with asthma development from some perspectives. There is no doubt that leptin plays a pro-inflammatory role in obese asthma. Certain investigations are needed to explore the mechanisms of leptin in the complex process of asthma development and other phenotypes of asthma.

## Figures and Tables

**Table 1:** Correlations of leptin and asthma in clinical cohort studies, ↑ = increased level; ↓ = decreased level.

Study	Type	Study Population	Directionality of Asthma-Leptin Relation	Main Results
[72]	Case-control	25 asthmatic pediatric patients and 10 controls aged from 6 to 18	Higher leptin in patients with asthma	↑ leptin in serum in asthmatic subjects vs. healthy controls
			↑ leptin parallel to asthma exacerbation	↑ leptin in asthma exacerbation period vs. in the asymptomatic period
[89]	Cross-sectional	62 symptomatic seasonal allergic rhinitis (SAR) patients in season	direct direction with allergy symptoms	Higher leptin in symptomatic female patients compared to normal subjects
		41 symptomless SAR patients out season, and 34 controls		Higher leptin in symptomatic male patients compared to symptomless and normal subjects
[88]	Cross-sectional	21 obese (OA) and 14 with non-obese asthma (NOA)	Indirect direction with weight loss and lung function	Leptin showed a negative relationship with FEV1 (%)
		35 obese (O) patients, and 33 controls (HC)		
[87]	Cross-sectional	30 women with asthma	Higher leptin in overweight patients with asthma	↑ leptin in serum in overweight asthma patients compared to normal weight patients
[86]	Cross-sectional	41 obese women with asthma and 40 non-obese women with asthma	Higher leptin in obese patients with asthma	↑ leptin in serum in obese patients with asthma compared to nonobese patients with asthma
			In correlation with BMI	Positive relationship between body mass index (BMI) and serum leptin levels
[90]	Cross-sectional	90 asthmatic women	↑ leptin parallel to asthma severity	Serum leptin correlated positively with asthma severity
			Inverse direction with lung function	Serum leptin correlated inversely with FEV1 and FVC
[94]	Cross-sectional	28 patients with asthma (BMI ≥ 25 kg/m2), 26 controls (BMI ≥ 25 kg/m^2^)	↑ leptin parallel to BMI and waist circumference	A significant relation between leptin concentration with BMI and waist circumference
		26 patients with asthma (BMI < 25 kg/m^2^)	No correlation of asthma	No significant difference of leptin level between overweight asthma patients and overweight healthy controls
[95]	Cross-sectional	80 women with obesity (grade II and III) asthma	No association between leptin and lung function	No difference between leptin in sputum or blood and FVC, FEV1, FEV1/FVC
[96]	Cross-sectional	65 patients with asthma, aged 2 to 14 yrs	↑ leptin parallel to asthma severity	Leptin levels positively correlated with the asthma severity
			No relationship with BMI	No correlation between leptin and BMI
[100]	Cross-sectional	122 children with asthma	↑ leptin parallel to asthma severity grades	Leptin was positively correlated with the disease grades in asthma children
[92]	Longitudinal	35 female patients with asthma	Indirect direction with lung function	The serum level of leptin was positively correlated with asthma symptom score
				The serum level of leptin was negatively associated with lung function
[91]	Longitudinal	84 postpubertal obese adolescents	Indirect relation with lung function	↓ leptin in parallel with improvements in FVC, FEV1 and PEF
			Indirect association with weight loss	↓ leptin in serum after weight loss
[93]	Longitudinal	19 asthma patients with bariatric surgery (Roux-en-Y gastric bypass)	no association of lung function with leptin	Significant reductions in the serum levels of leptin in bariatric surgery over time
			reducing leptin over time	No significant correlation between leptin and lung function.
[99]	Longitudinal	23 children with mild-to-moderate, newly diagnosed asthma	Higher leptin before budesonide treatment	↑ leptin before budesonide treatment after budesonide treatment and vs. control group
				Serum leptin levels correlate positively with body mass indices after budesonide treatment
[101]	Longitudinal	10 asthma patients with BMI > 30 and less than 300 pounds	↓ leptin after calorie restriction days	↓ serum leptin after calorie restriction days compared to ate ad libitum days

**Table 2:** Cellular mechanisms of leptin in immune responses, ↑ = increased level; ↓ = decreased level.

Cell	Cellular Mechanism of Leptin	Cellular Effect	General Effecct	Reference
LymphocyteTh1		↑ IFN-γ production	proinflammatory	[119]
		shifts T-helper (Th) cells to Th1 cells, ↑ IFN-γ	proinflammatory	[126]
LymphocyteTh2		↑ IL-4 and IL-10	Anti-inflammatory	[120,121]
		↑ IL-4, IL-5, and IL-13 under a type 2 condition	proinflammatory	[122]
Treg cells		↓ Foxp3 (+) CD4 (+) CD25 (+)	↓ Treg cells	[121]
Th17 cells	RORγt	↑ IL-17	Th17 responses↑	[124]
		↑ IL-4^−^IL-17^+^IFN-γ^-^		[125]
monocytes/ macrophages	obR long-form receptor and PI3K	chemoattract monocytes/macrophages	proinflammatory	[131]
		↑ phagocytic function of macrophages/monocytes, and ↑ leukotriene synthesis in pulmonary K. pneumoniae infection	proinflammatory	[132]
		↑ inflammatory cytokines (TNF-α, IL-6), ↑ reactive oxygen species	proinflammatory	[76]
		↑ TNF-α and IL-6	proinflammatory	[133,134]
		↑ proliferation, ↑ cytokine secretion (IL-6 and TNF-α).	proinflammatory	[135,136]
		↑ IL-6 and TNF-α in PBMC	proinflammatory	[138]
neutrophils		↑ neutrophil chemotaxis	↑ neutrophil at inflammatory foci	[144]
	ERK1/2 and p38-MAPK	↑ neutrophil chemotaxis	↑ neutrophil at inflammatory foci	[112]
	MEK1/2 and NF-κB	↓ neutrophil death	↑ neutrophil at inflammatory foci	[147]
	obR short-form receptor	↓ neutrophil apoptosis at high concentrations in vivo and in vitro	↑ neutrophil at inflammatory foci	[131,149]
		↑ bacterial phagocytic function	proinflammatory	[150]
eosinophils	calcium mobilization	↑ eosinophil chemotaxis	↑ eosinophil at allergic inflammatory foci	[112]
	ob-Rb	↓ eosinophil spontaneous apoptosis	↑ eosinophil at allergic inflammatory foci	[151]
	MAPK	↑ eosinophil migration, ↑ IL-1β, IL-6, IL-8, GRO-α and MCP-1	Proinflammatory	[155]
dendritic cells		↑ TNF-α, IL-1β, IL-6, and MIP-1α	Proinflammatory	[157]
	STAT3-HK2	↑ glycolytic metabolism	Proinflammatory	[159]
ILC2s		↑ proliferation	↑ ILC2s in the lung	[122]
mast cells	intracellular Ca^2+^ and chemokine CCL3	↑ degranulation and histamine	proinflammatory	[163]
